# Transcriptomics and Spatial Proteomics for Discovery and Validation of Missing Proteins in the Human Ovary

**DOI:** 10.1021/acs.jproteome.3c00545

**Published:** 2023-12-12

**Authors:** Loren Méar, Xia Hao, Feria Hikmet, Pauliina Damdimopoulou, Kenny A. Rodriguez-Wallberg, Cecilia Lindskog

**Affiliations:** † Department of Immunology, Genetics and Pathology, Cancer Precision Medicine Research Program, Uppsala University, Uppsala 751 85, Sweden; ‡ Division of Obstetrics and Gynecology, Department of Clinical Science, Intervention and Technology, Karolinska Institutet, Stockholm 14186, Sweden; § Department of Gynaecology and Reproductive Medicine, Karolinska University Hospital, Stockholm 171 77, Sweden; ∥ Department of Oncology-Pathology, Laboratory of Translational Fertility Preservation, Karolinska Institutet, BioClinicum, Stockholm 171 64, Sweden

**Keywords:** missing proteins, ovary, oocyte, ovarian follicle, fertility, antibody-based proteomics, immunohistochemistry, bulk RNA, multiplexed immunofluorescence, HPA

## Abstract

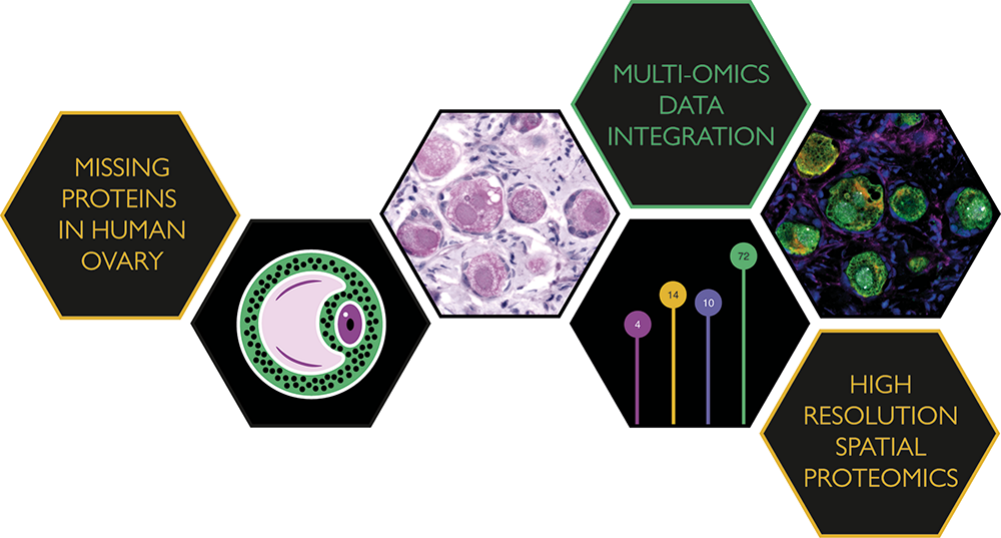

Efforts to understand the complexities of human biology encompass multidimensional aspects, with proteins emerging as crucial components. However, studying the human ovary introduces unique challenges due to its complex dynamics and changes over a lifetime, varied cellular composition, and limited sample access. Here, four new RNA-seq samples of ovarian cortex spanning ages of 7 to 32 were sequenced and added to the existing data in the Human Protein Atlas (HPA) database www.proteinatlas.org, opening the doors to unique possibilities for exploration of oocyte-specific proteins. Based on transcriptomics analysis of the four new tissue samples representing both prepubertal girls and women of fertile age, we selected 20 protein candidates that lacked previous evidence at the protein level, so-called “missing proteins” (MPs). The proteins were validated using high-resolution antibody-based profiling and single-cell transcriptomics. Fourteen proteins exhibited consistent single-cell expression patterns in oocytes and granulosa cells, confirming their presence in the ovary and suggesting that these proteins play important roles in ovarian function, thus proposing that these 14 proteins should no longer be classified as MPs. This research significantly advances the understanding of MPs, unearthing fresh avenues for prospective exploration. By integrating innovative methodologies and leveraging the wealth of data in the HPA database, these insights contribute to refining our understanding of protein roles within the human ovary and opening the doors for further investigations into missing proteins and human reproduction.

## Introduction

Efforts to decode the foundational components of human existence span across multiple levels. An increasing number of global initiatives are aiming to thoroughly map genes and proteins. Proteins play indispensable roles in orchestrating biological processes, and characterizing their expression and function is profoundly necessary to enhance our insights into human health.

The Human Proteome Project (HPP) is an international initiative led by the Human Proteome Organization (HUPO), with the primary goal of comprehensively characterizing and understanding the human proteome.^1^ Accordingly, the neXtProt knowledge database (www.nextprot.org) updates its Protein Existence (PE) classification every year.^2^ In the latest update (2023–04–18), 90.23% of all proteins predicted by the human genome have undergone empirical validation (mainly by mass spectrometry (MS) methodologies), thus being designated as PE1 proteins (*n* = 18,397). The absence of substantiated evidence at the protein level, however, leaves a subset of proteins called missing proteins (MPs). The MPs can be classified into three types: those with transcript evidence (PE2, *n* = 1151), those based on homology (PE3, *n* = 215), and those predicted (PE4, *n* = 15). This lack of evidence may be due to factors such as low expression levels, presence in transitory cell states, or expression restricted to rare cells that are challenging to sample or only represent a small proportion of the cells in a tissue. As a result, identifying the cells and tissues in which these MPs are expressed poses a significant challenge for revealing their identity and characteristics.

The Human Protein Atlas (HPA) is a comprehensive database that systematically maps human protein expression across all major tissues and cell types. Based on an integrated omics approach combining quantitative information from transcriptomics with spatial information based on stringent antibody-based imaging, the open-access database www.proteinatlas.org constitutes an important resource for understanding human biology and disease by untangling previously unknown patterns of protein localization, distribution, and abundance. Due to its comprehensive coverage of protein expression across diverse tissues and cells, the HPA will be able to play a crucial role in the quest for MPs, providing a valuable repository of data that can be used to identify and validate these elusive proteins.

The ovary stands as one of the most dynamic organs in humans, playing a pivotal role in both the endocrine and reproductive systems.^3^ During the reproductive years, spanning from puberty to menopause, the ovary fulfills dual functions: hormone production and the monthly release of a mature oocyte. The meiosis is initiated already during fetal development and comes to halt at the diplotene stage of prophase I. The meiotic progression remains suspended for several years, until puberty. At this stage, the influence of pituitary gonadotropins prompts the oocytes to reenter meiosis I, leading to an intermediate phase, known as metaphase I. Ultimately, a dominant oocyte will reach the metaphase II stage and can be ovulated. The oocyte, surrounded by granulosa cells, shapes a unique and functional structure: the follicle. The nongrowing follicles are situated within the ovarian cortex, the ovary’s outer layer. As follicles grow, they migrate into the medulla, the inner region of the ovary. Folliculogenesis can reach completion solely after the onset of puberty, culminating in ovulation, which is the release of the metaphase II oocyte. The count of follicles is finite and dramatically declines from birth to puberty, with only a handful proceeding through the entire folliculogenesis process. For most follicles, the natural end is death through atresia. Given the restricted number of follicles, their disparate distribution throughout the tissue, the dynamic shift in cellular composition over a woman’ life and menstrual cycle, and the inherent complexities involved in obtaining appropriate ovarian samples (from healthy prepubertal and reproductive-age women), the proteome of the immature oocyte remains insufficiently comprehended.

In the standard HPA workflow, the ovary is one of the tissue types that have undergone profiling using both bulk mRNA sequencing (RNA-seq) and immunohistochemistry (IHC).^4^ Until now, the transcriptomics data has been based on a consensus data set consisting of samples sequenced as part of the HPA consortium as well as data from samples collected within the GTEx consortium.^5^ All HPA samples (*n* = 3) and the majority of samples from the GTEx consortium (*n* = 107 out of 180) were from potential postmenopausal women (50–80 years old), resulting in an exceedingly rare number of follicles within the analyzed samples. As a result, genes specifically expressed in follicles often fell under the detection limit, posing challenges in identifying novel not previously described proteins potentially expressed in these rare cells. Last year, we published a case study in which the HPA was used to validate the expression of seven MPs in the human ovary.^6^ This validation was achieved by comparing data obtained through antibody-based proteomics and single-cell RNA sequencing (scRNA-seq). Out of these seven proteins, two are no longer classified as MPs: MRO and ZNF793. As part of the present investigation, four new RNA-seq samples of ovarian cortex spanning ages of 7 to 32 have been sequenced and added to the HPA data set, with the data accessible in the most recent version 23.0 of the HPA database, opening the doors to unique possibilities for exploration of oocyte-specific proteins. Our study aims to identify and validate MPs in the human ovary, with a specific focus on immature follicles. Based on transcriptomics analysis of the four new tissue samples representing both prepubertal girls and women of fertile age, we have selected candidates for antibody-based profiling using the same tissue samples, constituting a unique possibility of validating tissue-specific expression patterns with different methodologies. Finally, to confirm the cell type specificity, the results have been compared with single-cell transcriptomics data and the novel candidates were further analyzed using multiplexed immunofluorescence.

## Experimental Procedure

### Human Tissue Samples and Tissue Preparation

Frozen ovarian cortex samples were collected from four individuals aged 7, 16, 20, and 32 years for fertility preservation and stored in liquid nitrogen. The samples were anonymized, and no clinical information except for the age was available. Samples were embedded in optimal cutting temperature (OCT) compound for cryo-sectioning and RNA isolation or fixed in fresh 4% paraformaldehyde (PFA) solution for histology and protein analysis overnight. Tissues were then dehydrated, starting with 70% alcohol, and impregnated with paraffin in 60 °C using a tissue embedding system (Tissue Processing Center TCP 15, MEDITE Medical GmbH, Germany). The tissues were embedded in paraffin blocks and routine 4 μm-thick microtome sections were cut, placed on glass slides (SuperFrost Plus, Menzel-Glaser, Germany), and stored at −20 °C. One control slide was stained with hematoxylin and eosin for tissue quality control.^7^ Furthermore, to optimize IHC staining, an anonymized human ovarian cortex tissue sample was obtained from a gender reassignment patient (30 years old) at Karolinska University Hospital. A written and oral informed consent form was signed by the patient in accordance with the Declaration of Helsinki. The tissue was retrieved from the operating room and transported to the research laboratory in PBS within 15 min. A portion of the cortex was removed from the medulla and was fixed in formalin and stored in paraffin. The project was approved by the Swedish ethical review authority nos. 2917/2124-31/3, 2020-05940, 2015/798-31/2, and 2021-04563.

### RNA Extraction and Sequencing

For total RNA isolation, the RNeasy Mini Kit (QIAGEN N.V., Venlo, Netherlands) was used as previously described.^4^ In brief, up to ten 15 μm sections were collected into a tube and lysed with lysis buffer and beta-mercaptoethanol. The tissue was homogenized mechanically with two metal beads and vortexed at maximum speed for 10–20 s. The solution was transferred to a spin column followed by a series of centrifugations according to the manufacturer’s description. RNA concentration was initially controlled with a NanoDrop spectrophotometer (Thermo Fisher Scientific, Waltham, Massachusetts), and RNA integrity was analyzed by the Agilent 2100 Bioanalyzer system (Agilent Biotechnologies, Palo Alto, USA) with the RNA 6000 Nano LabChip Kit. Only samples of high-quality RNA (RNA integrity number ≥7) were sequenced. The samples were prepared with the TruSeq PolyA selection kit and sequenced on a NovaSeq 6000 (Illumina Inc., San Diego, California), similarly as previously described.^8^ Transcript-normalized expression (“nTPM”) values for each protein-coding gene and sample were obtained following the HPA-standardized data analysis protocol as previously described.^4^ The data was based on The Human Protein Atlas version 23.0 and Ensembl version 109.

### Transcriptomics Data Analysis (Deconvolution)

To identify the most suitable deconvolution method for our data set, we used the R package “granulator” (https://github.com/xanibas/granulator), which enabled us to conduct benchmarking and estimate the proportions of four ovarian cell types (oocytes, granulosa cells, endothelial cells, and stromal cells) present in the four samples sequenced with RNA-seq. A reference profile was created by downloading single-cell type RNA-seq data from the HPA Web site v23.proteinatlas.org, processed as described previously.^9^ This data set included the nTPM value for each gene across 81 distinct cell types from 31 data sets. Subsequently, the cell types of interest—oocytes, granulosa cells, endothelial cells, and ovarian stromal cells—were selected from this data set. While the expression data for endothelial cells stems from the integration of all “endothelial cell” clusters across various data sets and tissues in the human body, those for oocytes, granulosa cells, and ovarian stromal cells are directly sourced and specific to the “ovary” data set (originally published by Wagner et al.^3^). To aid in benchmarking of the different deconvolution methods included in “granulator”, we have also estimated the proportion of cell types in the four samples by image analysis. Multiple deconvolution methods can also be evaluated with this package. Cell types were estimated by first covering the entire tissue area with squares at approximately 40,000 μm^2^ for each square on H&E-stained sections. Representative regions of interest (ROIs) with three main phenotypes—oocyte and granulosa cell-rich, endothelial cell-rich, or general ovarian stroma—were selected for each sample. QuPath Cell Detection was used to count the total number of nuclei within each of the ROIs and manually phenotype cells to estimate the percentages of oocytes, granulosa cells, endothelial cells, and ovarian stroma cells. The remaining squares were compared with the ROIs to extrapolate and estimate the total number of cells and percentages of each cell type in the entire sample (Supplementary Figure 1). Based on the results of the benchmarking, “dtangle” was identified as the most suitable method for deconvolution^10^ and the estimated cell type proportions presented in this study were derived from the application of this method.

### IHC

Slides corresponding to one tissue section per analyzed antibody were baked overnight in 50 °C, deparaffinized in xylene, and rehydrated in graded alcohols (99.9, 95, and 80%) down to deionized water. Endogenous peroxidases were blocked using 0.3% hydrogen peroxide in 95% alcohol, and the heat-induced epitope retrieval (HIER) was performed in a decloaking chamber (Biocare Medical, Walnut Creek, California, USA) at 125 °C for 4 min while the slides were immersed in 1× Target Retrieval Solution, pH 6.0 (Agilent Technologies Inc., Santa Clara, California, USA). Slides were then cooled to approximately 90 °C before rinsing with deionized water and stored immersed in TBS+Tween wash buffer (Thermo Fisher Scientific, TA-999-TT, Waltham, Massachusetts, USA). All antibody stainings were performed at room temperature (RT) using the Autostainer 480S (Thermo Fisher Scientific) and staining kits from Epredia (Epredia UltraVision LP HRP kit and DAB Detection System, Breda, Netherlands) with HPA standard IHC staining protocol previously described.^7^ Primary antibodies and their dilution for the top candidates are listed in Table S2. Then, the slides were digitized using an Aperio AT2 slide scanner (Leica Biosystems, Baden-Wurttemberg, Germany) with a 20× objective. Each digitized tissue sample was manually annotated using the HPA Laboratory Information Management System (LIMS), for each of the four cell types: oocytes, granulosa cells, endothelial cells, and stromal cells. The principal annotation parameter was staining intensity, which was based on the following standardized HPA scale: not detected (or negative), weak, moderate, and high.^4^ In cases where the cell type was not present in the whole tissue section, no conclusions could be drawn regarding the protein expression and the parameter “not set” was selected. For the final candidates, the annotation results (intensity, quantity, localization, and the summary) are available in Table S3. Ultimately, one slide per sample per antibody was analyzed.

### Multiplex Immunofluorescence (mIF)

Slides were pretreated the same as described for IHC above. After HIER, the slides were exposed to a LED-light bleaching process immersed in a bleaching solution consisting of 0.2 M glycine, 1.5% hydrogen peroxide, and 1× TBS+Tween (Thermo Fisher Scientific, TA-999-TT), for 1 h in RT.

Slides were incubated using a fixed panel of five marker antibodies in a six-cycle antibody staining process with intermediary deactivation steps after each cycle in 90 °C for 20 min, immersed in 1× Target Retrieval Solution, pH 6.0 (Agilent Technologies), using a decloaking chamber (Biocare Medical) for antibody stripping. Full cycle information (panel markers, antibodies, dilutions, reagents, incubation times, OPAL fluorophores) is available in Table S4. One cycle of staining includes blocking, primary antibody incubation, antirabbit IgG (H+L) with horseradish peroxidase (HRP) polymer, and an OPAL fluorophore (Akoya Biosciences, Marlborough, Massachusetts, USA). All cycles were performed at RT and using the Austostainer 480S (Thermo Fischer Scientific) and the HRP kit from Epredia (Epredia UltraVision LP HRP kit). After the last cycle, slides were incubated with the OPAL 780 fluorophore-conjugated anti-DIG antibody and 4′,6-diamidino-2-phenylindole (DAPI) (Invitrogen, D1306, Thermo Fisher Scientific). Slides were then mounted using Prolong Glass Antifade mounting medium and left to cure overnight in RT after which they were digitized using PhenoImager (Akoya Biosciences). Spectral unmixing and export of images were performed using the built-in spectral library of the inForm software (Akoya Biosciences). Ultimately, a single slide per sample was analyzed for the specified MPs.

### R Session Information

All the data processing and visualization were performed on R (version 4.2.2) and by using the following packages: data.table (version 1.14.8), dplyr (version 1.1.2), forcats (version 1.0.0), ggh4x (version 0.2.5), ggplot2 (version 3.4.2), ggpubr (version 0.6.0), ggridges (version 0.5.4), granulator (version 1.6.0), gridExtra (version 2.3), hrbrthemes (version 0.8.0), purr (version 1.0.1), readr (version 2.1.4), readxl (version 1.4.3), stringr (version 1.5.0), tibble (version 3.2.1), tidyr (version 1.3.0), tidyverse (version 2.0.0), viridis (version 0.6.4), and viridisLite (version 0.4.2).

### Data Availability

The four new sequenced ovarian tissue samples results are publicly available on the Protein Atlas version 23.0 (released 2023–06–19).

## Results

### Transcriptomic Analysis of Novel Ovary Samples

**Figure 1:**
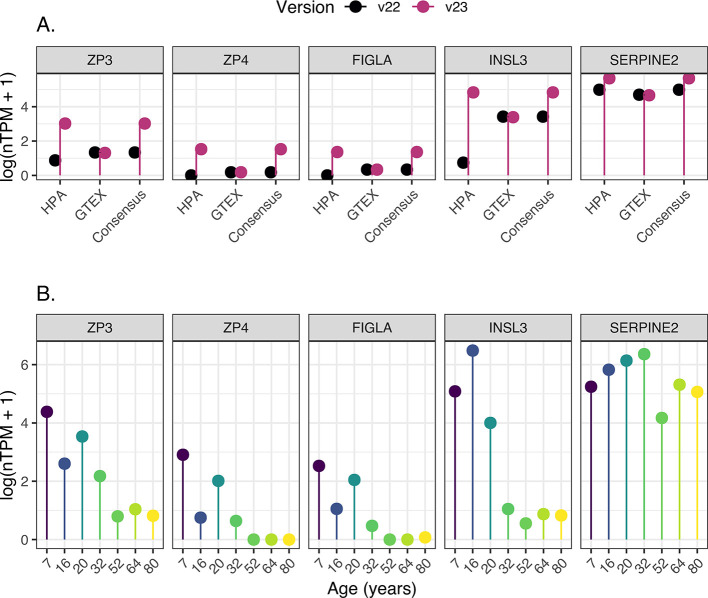
Log-normalized transcriptomic expression of genes newly identified as elevated in the ovary. These genes include well-known markers for oocytes (ZP3, ZP4, and FIGLA), theca cells (INSL3), and (SERPINE2). (A) A comparison between the prior version of the Human Protein Atlas (HPA) data set (v22—shown in black) and the latest version (v23—shown in purple). The visualization displays the average expression (log(nTPM+1)) across all individual samples in both HPA (v22, *n* = 3; v23, *n* = 7) and GTEx (*n* = 180), alongside the consensus value. (B) Expression values (log(nTPM+1)) across HPA samples (*n* = 7). Among these, the four youngest samples (ages: 7, 16, 20, and 32) were newly added in v23 of the HPA database, while the other three (ages 52, 64, and 80) were present in previous HPA versions.

Fresh frozen samples of ovarian cortex from four individuals (7, 16, 20, and 32 years old) were sequenced, and the data was processed using the same pipeline as the previous samples in the HPA database. The new HPA ovary data set now consists of seven samples out of which four were added as part of this study. Based on a normalization taking into consideration also the GTEx ovary data set (180 individuals), we were able to determine how many genes show an elevated expression in the ovary from a body-wide perspective. Interestingly, with the addition of the four new samples, 178 genes were elevated in the ovary in comparison to 35 other normal organs, representing 57 newly elevated genes compared to the previous data set, which is a striking number considering that the classification is based on the average expression in the ovary taking into account all the other HPA and GTEx samples. Among the newly elevated genes are, e.g., ZP3, ZP4, and FIGLA (oocyte markers), INSL3 (theca cells), and SERPINE2 (granulosa cells). This suggests that addition of the four new samples adds important insights on genes and proteins relevant for ovarian function. This was also confirmed by comparing the expression levels of these genes between the old and new data sets, where it is evident that genes associated with follicular cells show a significantly higher expression in the new data set (Figure 1a). SERPINE2 is also expressed by stromal cells, which contributes to the less pronounced difference in expression between the new and old data (Figure 1). The data also shows interindividual differences between the four new samples, with consistently lower expression levels of oocyte and granulosa cell-specific genes in the sample from the 32-year-old woman, indicating that this sample contains a smaller proportion of follicles (Figure 1b). Nevertheless, the expression levels of oocyte and granulosa cell-specific genes in this sample were higher than in samples from older women; thus, all four samples were therefore included in the present investigation.

### Estimations of Cell Type Proportions in Ovarian Samples Based on Deconvolution and Image Analysis

**Figure 2:**
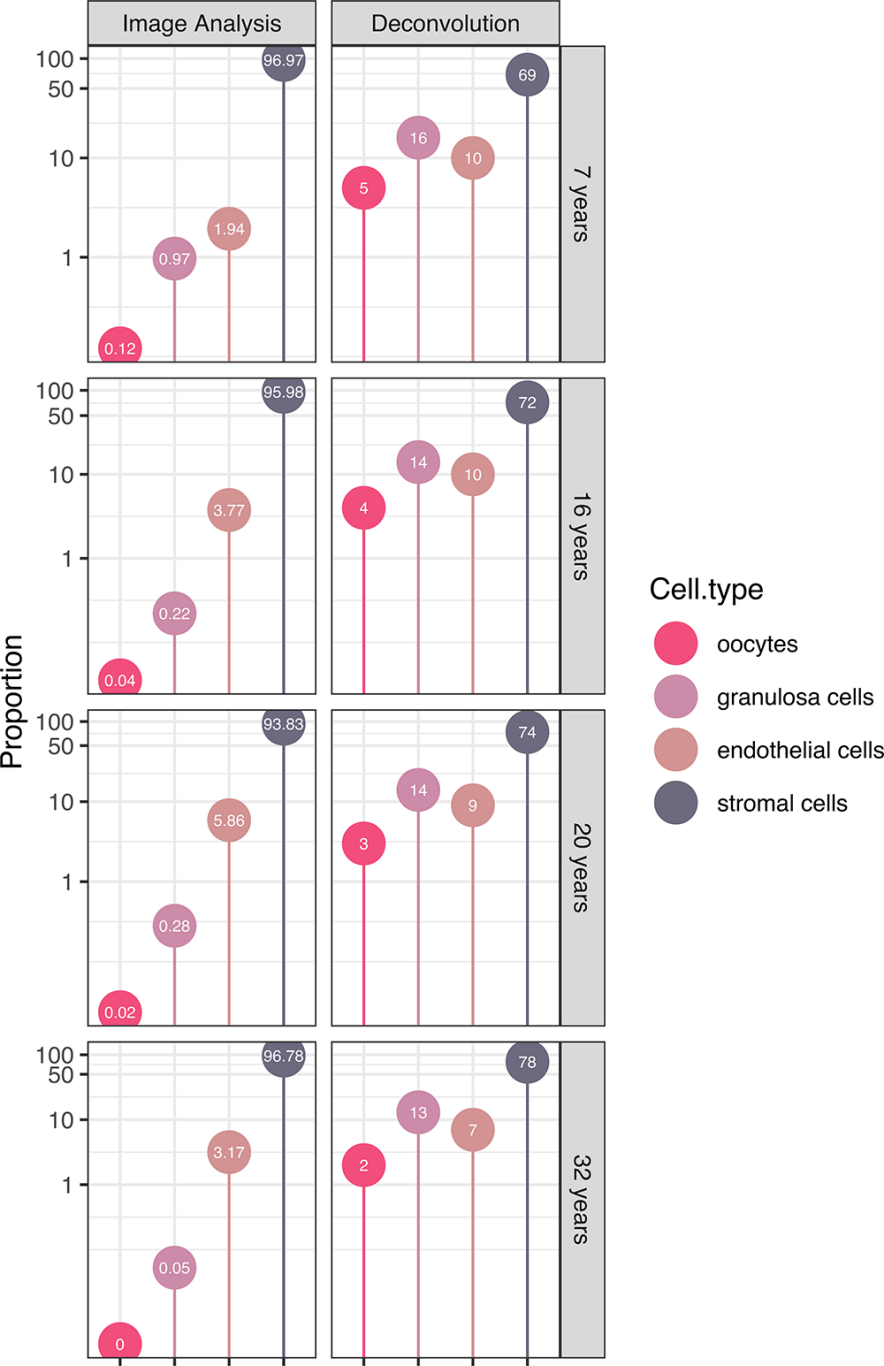
Estimation of the ovarian cell proportions (in percentage) utilizing automated image analysis (left panel) and bulk-RNA-seq data deconvolution (right panel) for the four youngest patients (ages: 7, 16, 20, and 32). One slide per patient was employed for automated image analysis. The analysis focused on four primary cell types: oocytes (vivid pink), granulosa cells (light lavender), epithelial cells (antique ruby), and stromal cells (slate gray).

Since bulk RNA-seq data is obtained from heterogeneous tissue samples with variable cellular compositions that may constrain analysis, we next aimed to estimate the proportion of cell types present in the samples used for RNA-seq. In recent years, many computational approaches have been developed to estimate cellular proportions in bulk RNA-seq data, such as deconvolution. By using a reference data set of single-cell expression profiles from women of reproductive age presented in the HPA database, we benchmarked different methods for deconvolution and created a framework for analysis of our four novel samples. The results from the deconvolution of the bulk RNA-seq data are presented in Figure 2, along with estimated cell type proportions generated by automated image analysis. In this study, we focused on four main cell types (oocytes, granulosa cells, epithelial cells, and stromal cells) while excluding others such as immune cells. The estimations of the cell type proportions appear to be overestimated within the deconvolution framework but underestimated through image analysis-based estimation (Supplementary Figure 1). It should be noted that tissues are not composed of a fixed and predetermined number of distinct cell types and cell types can display variations and subtypes that may not be adequately captured by either of the methods employed. Reference profiles based on single-cell transcriptomics data could not encompass the diverse stages of follicle development, including primordial and primary stages. On the other hand, image analysis-based estimation was performed on a single section from the same sample used for bulk RNA-seq analysis. While this approach offers insights into tissue quality and morphology, it may not accurately reflect the actual number of sequenced cells, thus lacking full representativeness.

The proportion of oocytes found in the ovarian cortex across our four samples varied between 2 and 5% when estimated based on deconvolution and between 0.004 and 0.121% when estimated through image analysis (Figure 2). This is consistent with data previously described by Wagner et al.,^3^ the adult ovary data set used as a reference profile, which suggested a very low proportion of oocytes (0.2%). The highest proportion was consistently observed in the sample from the 7-year-old individual, the youngest among the samples (Figure 2). Nevertheless, all results lead to the same overall conclusion: the predominant component in the ovarian tissue samples remains the stromal cells, succeeded by endothelial cells, granulosa cells, and finally, oocytes, further highlighting the necessity of corroborating transcriptomics findings through spatial protein expression profiling.

### Target Genes and Candidate Selection

**Figure 3:**
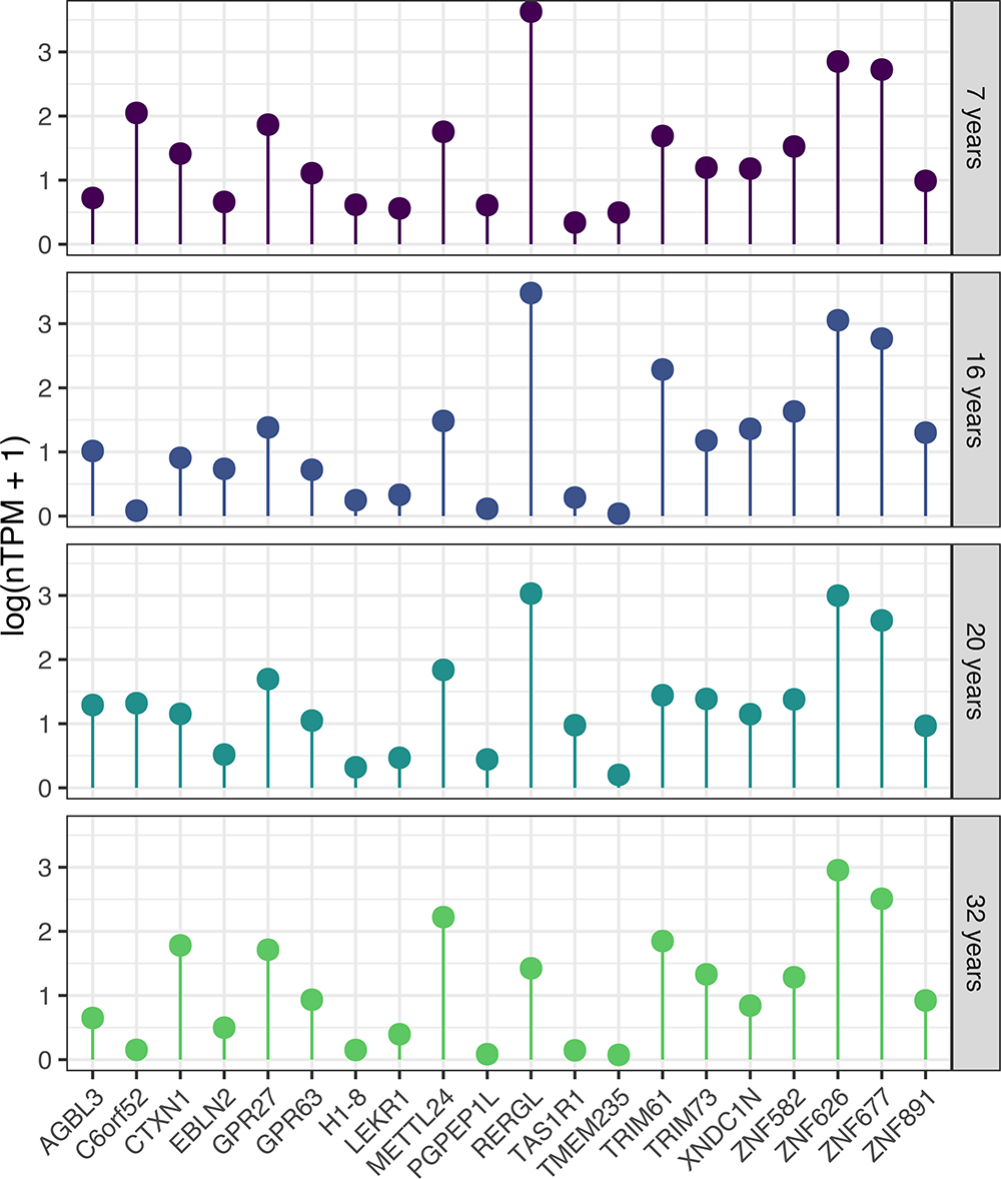
Log-normalized transcriptomic expression (log(nTPM+1)) of the 20 candidate MPs across the four samples, each representing one patient (ages: 7, 16, 20, and 32).

Next, we aimed to identify MP candidates suitable for an extensive spatial analysis in the four new tissue samples. Based on the most recent neXtProt update (2023–04–18), a total of 1381 proteins are still categorized as MPs, delineated into 1151 PE2, 215 PE3, and 15 PE4. Out of these 1381 MPs, 269 MPs (248 PE2 and 21 PE3) have antibody data published in the most recent version of the HPA database, excluding antibodies targeting sequences with high homology toward other human proteins (multitargeting antibodies). To identify protein candidates potentially expressed in follicles, we generated a new data set of transcriptomics levels only in the four new samples. Based on the same filtration process as our previous study^6^ using an expression threshold of 0.5 nTPM in at least one of the four samples, a total of 14,367 out of the 20,162 genes remained. Among these 14,367 genes, 61 out of the 269 MPs with antibodies available in the HPA remained (detailed in Table S1), all classified as PE2. Out of the initial 61 candidates, 16 were excluded based on a thorough manual screening of the HPA database. Unfortunately, TTLL3, which has been described and validated previously, was excluded due to low antibody volume. Additionally, SEM1, which possesses two distinct identifiers in UniProt/neXtProt, one PE1 and the other PE2, was also excluded since it is not possible to determine which of these two proteins is targeted by the antibody.^6^ Fourteen additional proteins were excluded taking into consideration available antibody volume and staining pattern in the ovary, e.g., lack of staining in follicles in present samples available in the HPA database, or unspecific antibody staining. Finally, 45 MPs were stained and optimized with IHC on a large section of ovarian cortex containing a significant number of follicles, out of which 20 final candidates (AGBL3, C6orf52, CTXN1, EBLN2, GPR27, GPR63, H1–8, LEKR1, METTL24, PGPEP1L, RERGL, TAS1R1, TMEM235, TRIM61, TRIM73, XNDC1N, ZNF582, ZNF626, ZNF677, and ZNF891) yielded distinct staining in follicles and were selected for in-depth IHC analysis. The mRNA expression levels of these 20 candidates are displayed in Figure 3. Compared with our prior publication,^6^ this study identified 35 MPs with available antibodies that were unidentified in last year’s study. Ten of these were stained in the present investigation (AGBL3, GPR27, H1–8, PGPEP1L, TAS1R1, TMEM235, XNDC1N, ZNF626, ZNF677, and ZNF891), while the remaining 25 were not selected due to suboptimal staining pattern during screening or IHC optimization. Twenty-six proteins were present in both the previous and this data set, out of which 10 were analyzed as part of this study (C6orf52, CTXN1, EBLN2, GPR63, LEKR1, METTL24, RERGL, TRIM61, TRIM73, and ZNF582). A total of 16 proteins were exclusively present in the previous paper. Seven of them are no longer classified as MPs (C11orf53, C1orf54, CYB5RL, FAM110D, MRO, RGL4, and ZNF793), six are incompletely described (ALG1L, C12orf76, QRFP, STRC, ZBED6CL, and ZNF781), and three do not meet the expression cutoff criteria in our new sample assessment (ANKRD61, KIF25, and PROX2).

### Protein Profiling of MPs in the Ovary

**Figure 4:**
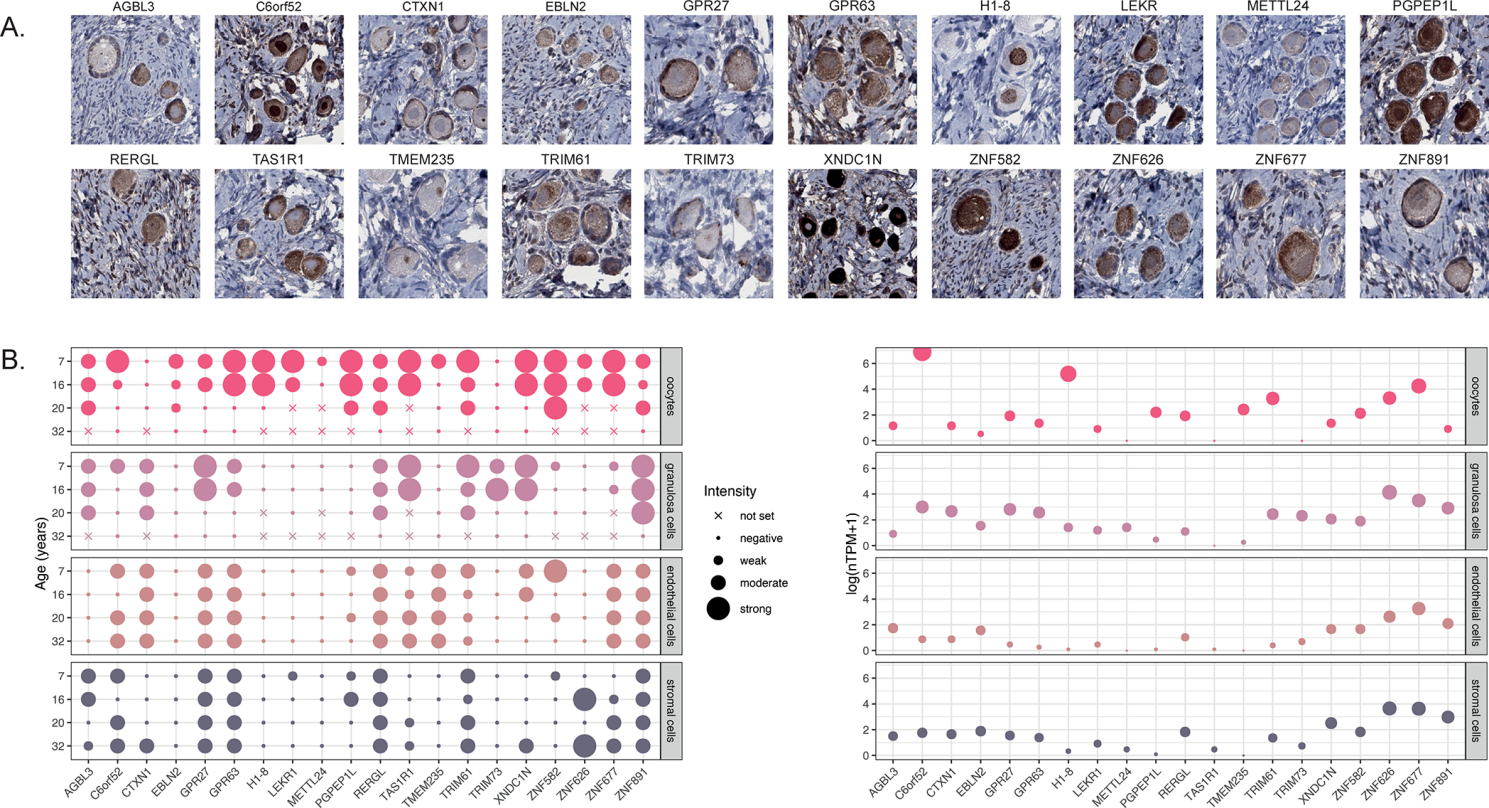
Protein profiling of the 20 candidate MPs in novel ovarian samples. (A) Representative images of IHC staining patterns of the 20 candidate MPs in human ovarian tissue from a 7-year-old individual. Brown staining corresponds to antibody binding, and all nuclei are stained with hematoxylin in blue as counterstaining. One whole tissue slide per patient (*n* = 4, ages: 7, 16, 20, and 32) per antibody (*n* = 20) was analyzed. (B) Overview of protein expression levels based on IHC staining intensity, as well as transcriptomics levels based on scRNa-seq from the HPA, originally from Wagner et al. Expression levels are shown in four cell types: (oocytes (vivid pink), granulosa cells (light lavender), epithelial cells (antique ruby), and stromal cells (slate gray)). Dot sizes correspond to staining intensity or scaled expression levels (log(nTPM+1)), while a cross indicates the absence of the cell type on the analyzed slide.

The 20 final candidates were stained with IHC on samples from the four new individuals used for RNA-seq analysis (antibody information available in Table S2), and the staining patterns were annotated manually (data shown in Table S3). Representative IHC images and an overview of the staining intensity across four ovarian cell types (oocytes, granulosa cells, endothelial cells, and stroma) are displayed in Figure 4, showing that the positivity observed in follicle cells in the test staining could be reproduced also in these novel samples for all 20 candidates. Ten of the analyzed proteins showed stronger expression in oocytes (C6orf52, EBLN2, H1–8, LEKR1, MELTTL24, PGPEP1L, TMEM235, ZNF582, ZNF626, and ZNF677), two were more highly expressed in granulosa cells (CTXN1 and TRIM73), and eight proteins showed staining of equal intensity in both oocytes and granulosa cells (AGBL3, GPR27, GPR63, RERGL, TAS1R1, TRIM61, XNDC1N, and ZNF891). While certain proteins appeared to be highly specific to follicle cells (EBLN2, H1–8, LEKR1, METTL24, PGPEPL1, TRIM73, and ZNF582), many proteins exhibited additional staining in endothelial cells (CTXN1, TA1SR1, TMEM235, XNDC1N), stromal cells (AGBL3, ZNF626), or both (C6orf52, GPR27, GPR63, RERGL, TRIM61, ZNF677, and ZNF891). Based on the IHC analysis alone, we cannot rule out whether positivity observed in endothelial cells and stromal cells represents true protein expression or nonspecific antibody binding.

### Orthogonal Validation of MPs Based on Single-Cell Transcriptomics

For orthogonal validation of the single-cell type expression patterns observed by IHC, we plotted the single-cell RNA expression levels of the 20 candidates using the reference profile from the deconvolution analysis (i.e., processed data from the HPA database, originated from Wagner et al.^3^ (Figure 4B). The analysis showed that for 14 genes (70%), similar expression profiles in the four cell types were observed using both IHC and scRNa-seq. Three genes were highly specific to follicle cells using both IHC and scRNA-seq: H1–8 and PGPEP1L expressed in oocytes and TRIM73 expressed in granulosa cells. Two genes (C6orf52 and TMEM235) showed an elevated expression in oocytes in both data sets but were more specific in the scRNA-seq data set, suggesting nonspecific antibody binding to endothelial and/or stromal cells. For nine genes (AGBL3, CTXN1, GPR27, GPR63, RERGL, TRIM61, ZNF626, ZNF677, and ZNF891), expression in either oocytes, granulosa cells, or both could be confirmed by IHC and scRNA-seq, but both methods also showed additional expression in endothelial and/or stromal cells, suggesting that these proteins are indeed expressed in multiple cell types within the ovary. Finally, six genes displayed discordant mRNA and protein expression patterns (EBLN2, LEKR1, METTL24, TAS1R1, XNDC1N, and ZNF582). In these cases, the IHC staining showed highest expression in either oocytes (EBLN2, LEKR1, METTL24, and ZNF582) or both oocytes and granulosa cells (TAS1R1 and XNDC1N), while the expression based on scRNA-seq was either low in general or more highly expressed in endothelial and/or stromal cells. This discrepancy may be attributed to the influence of age on expression levels, as certain proteins exhibit highly specific expression in the youngest samples, corresponding to immature follicles and oocytes. Consequently, the validation of their expression using scRNA-seq data from adult individuals becomes challenging, hindering conclusive interpretations for these proteins.

### Validation of MPs Based on Multiplex Antibody-Based Imaging

**Figure 5:**
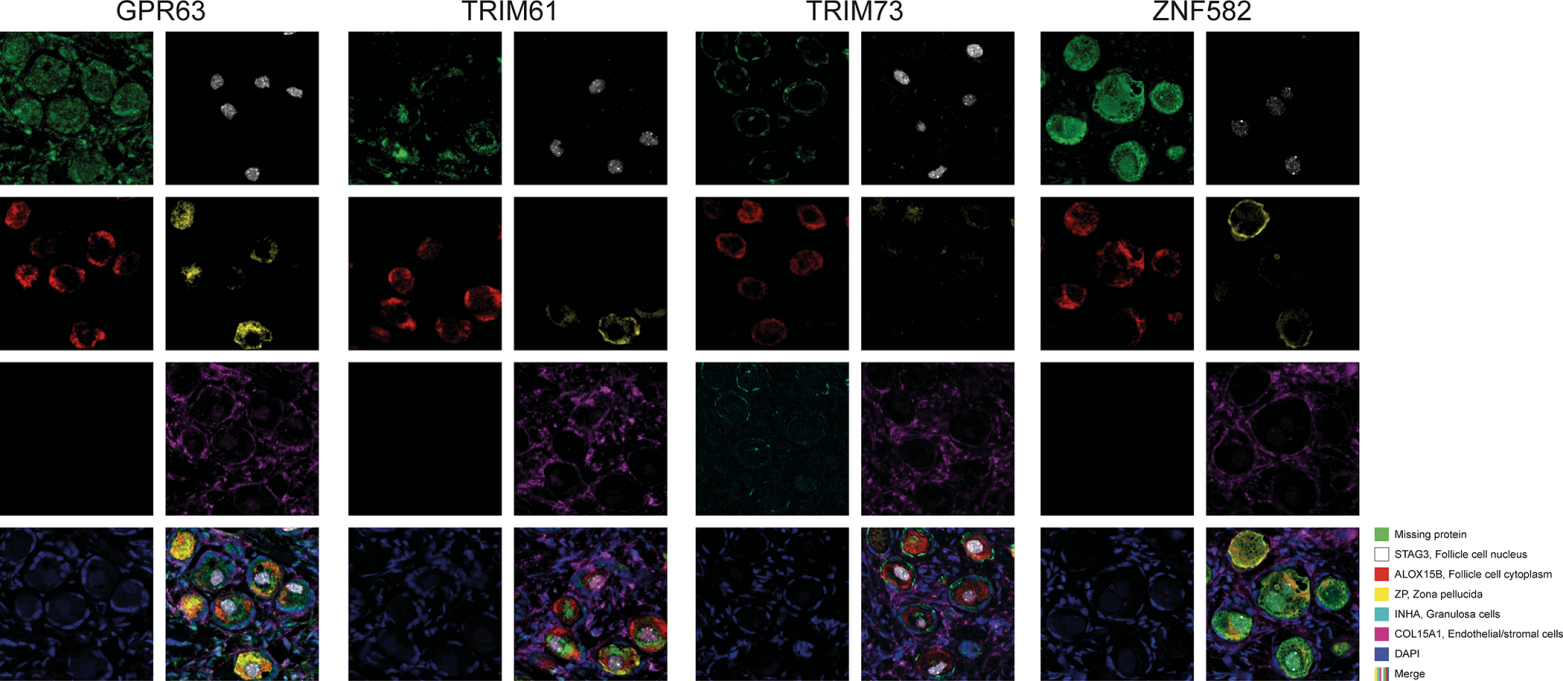
Representative images of multiplex immunofluorescence staining patterns of four selected MPs (GPR63, TRIM61, TRIM73, ZNF582) visualized in green, stained one by one together with a fixed panel of cell type-specific markers targeting follicle cell cytoplasm (ALOX15B, red), follicle cell nucleus (STAG3, white), granulosa cells (INHA, cyan), zona pellucida (ZP, yellow), endothelial and stromal cells (COL15A1, magenta), and a nuclear marker (DAPI, blue). The showcased images are derived from a single patient (age: 7).

While regular IHC constitutes a standard method for antibody-based proteomics, staining patterns in small subsets of cells may be challenging to determine by the human eye, and variations in staining intensity between cell types may be misinterpreted due to saturation of the staining. To establish a complementary antibody-based technology that may aid in interpretation and further validate the cell type-specific expression, four MP candidates with various technological challenges identified both in the previous^6^ and current studies (GPR63, TRIM61, TRIM73 and ZNF582) were selected for mIF imaging. A panel of five well-known markers targeting various cell types and structures within ovarian tissue was used for comparison with expression patterns of the selected MPs: ZP2, ALOX15B, and STAG3 for oocytes, INHA for granulosa cells, and COL15A for endothelial and stromal cells. The panel of five markers was stained together with the four selected MPs, one by one, based on a 6-plex immunofluorescence strategy, together with DAPI for outlining the nuclei. Unfortunately, the granulosa cell marker INHA failed when stained together with GPR63, TRIM61, and ZNF582. Representative immunofluorescence images are displayed in Figure 5.

GPR63 showed concordant results between IHC and scRNA-seq in follicle cells, along with substantial staining in endothelial and stromal cells using IHC. Immunofluorescence could have allowed for the mitigation of this effect due to a higher signal-to-noise ratio, but in this instance, the same result was obtained with both staining methods. Concerning TRIM61, oocytes and granulosa cells showed equivalent expression levels in the IHC analysis while scRNA-seq data pointed toward a slightly higher expression in oocytes. The multiplex experiment allowed us to refine the expression profile of this protein to be more highly expressed in oocytes. TRIM73 had been selected for multiplex analysis with the purpose of confirming its specific expression within granulosa cells. The IHC and RNA results had already yielded compelling evidence, yet the multiplex analysis enabled the specificity of this protein to be confirmed through the application of an alternative antibody-based methodology. IHC results for ZNF582 pointed toward a highly specific expression in oocytes, while the scRNA-seq data was more diffuse. The multiplex analysis confirmed the oocyte-specific expression observed by IHC.

## Discussion

The ovary constitutes a challenging tissue type in large-scale omics studies due to several reasons. The dynamic and complex biological processes involving maturation of oocytes not only affect gene and protein expression patterns throughout the menstrual cycle and during the life-span of a woman, but some events also take place already during fetal development. Access to representative tissue samples from healthy individuals with intact follicles remains a significant obstacle, and even if such samples are available, follicle cells only correspond to a small proportion of the cells in the tissue, making them difficult to analyze. Furthermore, as both the cell morphology and gene expression of the follicles vary with age and hormonal factors, studies based on a few samples will not be able to give a full understanding of the molecular repertoire of human follicle cells. Previous studies on ovary are underrepresented in the quest for MPs,^6^ a set of proteins that lack previous evidence of existence at the protein level as defined by the HPP consortium.^1^ Here, we used a unique collection of ovarian samples from both prepubertal girls and women of fertile age to investigate if any of the 1381 proteins defined as MPs could be confidently identified in human follicles.

In the present investigation, we took advantage of the extensive transcriptomics and antibody-based proteomics data sets publicly available in the HPA database, both to identify suitable candidates for IHC analysis by screening already validated antibodies and stained images of the human ovary, and to validate the newly generated data. Out of 269 genes with available antibody data in HPA v23, 61 genes showed expression in at least one of the four new ovary samples based on bulk RNA-seq, and 20 were finally selected for in-depth spatial proteomics analysis using antibody-based imaging.

It is reassuring that all 20 analyzed proteins exhibited distinct staining in either oocytes, granulosa cells, or both using the new samples, and as many as 14 proteins (70%) showed consistent results at the single-cell type level when compared with data from scRNA-seq, thereby suggesting that these 14 proteins (AGBL3, C6orf52, CTXN1, GPR27, GPR63, H1–8, PGPEP1L, RERGL, TMEM235, TRIM61, TRIM73, ZNF626, ZNF677, and ZNF891) are indeed expressed in human ovarian tissue. Three proteins are particularly interesting as they based on both IHC and scRNA-seq showing an elevated expression in either oocytes (H1–8 and PGPEP1L) or granulosa cells (TRIM73), together with very low or absent expression in surrounding endothelial or stroma cells.

H1–8, also known as H1FOO, regulates gene expression by influencing the arrangement of chromatin structures and has previously been identified through rt-PCR in human oocytes in 2003,^12^ suggesting a role in oogenesis and early embryogenesis. In 2022, a study affirmed the involvement of H1–8 in regulating oogenesis in mice.^13^ Another research group, however, suggested that H1–8 may not be essential for mouse oogenesis and fertility.^14^ It has been established that H1–8 is necessary for bovine subjects during the preimplantation stage.^15^ More recently, a review concluded that H1–8 is activated during the germinal vesicle stage, effectively replacing core histone H1 until the oocyte reaches full maturity (MII) in mice.^16^ A study published in 2023^17^ demonstrated a correlation between H1–8 expression and oocyte maturity in humans. Until now, its presence at the protein level has not yet been confirmed, but here we present for the first time convincing evidence of existence for H1–8 protein expression in human oocytes. PGPEP1L, the second protein with robust expression restricted to oocytes confirmed by the scRNA-seq data, is an enzyme predicted to be involved in proteolysis. No previous study has described PGPEP1L in relation to the ovary, but interestingly, PGPEP1L also shows expression in human testis in the HPA database both using IHC and bulk RNA-seq, suggesting a function related to reproduction. Here, we confidently show that PGPEP1L is expressed in oocytes. TRIM73, which showed an elevated expression in granulosa cells confirmed by both IHC and scRNA-seq, has not been previously described in relation to normal human tissues except for in our previous study.^6^ Since this protein was identified in granulosa cells using two different strategies for candidate selection and the analysis was performed on different samples, we believe that TRIM73 plays a role in granulosa cells of the human ovary. To further support our findings, the same results were obtained based on mIF, confirming overlap with the granulosa cell-specific marker INHA.

Two proteins (C6orf52 and TMEM235) that showed an elevated expression in oocytes with both data sets but were more specific in the scRNA-seq data set should be further validated by additional experiments in order to confirm their oocyte specificity at the protein level. It is possible that the additional positivity to endothelial and/or stromal cells is caused by nonspecific antibody binding, which can be ruled out by testing other antibodies toward the same proteins, or analyzing these targets by other methods. It should also be noted that IHC analysis is only semiquantitative and the staining becomes saturated, which may suggest that the difference in protein expression levels between oocytes and other surrounding cells is larger than reflected by differences in staining intensity. Nevertheless, both IHC and scRNA-seq suggest that these two proteins are expressed in human oocytes, and except for C6orf52 also being identified in our previous study, neither of these proteins have previously been described in human tissues or in the context of ovary.

Nine proteins (AGBL3, CTXN1, GPR27, GPR63, RERGL, TRIM61, ZNF626, ZNF677, and ZNF891) showed expression in follicle cells according to IHC and scRNA-seq, but both methods also showed additional expression in endothelial and/or stromal cells. Four of these proteins (CTXN1, GPR63, RERGL, and TRIM61) were identified also in our previous study, suggesting a potential role in the human ovary, and interestingly, two have previously been linked to reproductive function in the literature. GPR63 has been associated with egg production in ducks,^18^ and the response of the RERGL gene was altered in irradiated perch gonads.^19^ While the staining of nine proteins was not restricted to oocytes or granulosa cells only, all these candidates were distinctly expressed in follicle cells based on both methods and it is still possible that these proteins possess ovary-specific functions despite lower levels of expression being observed in other surrounding cell types. The large proportion of endothelial and stromal cells in the human ovary plays important roles in supporting oogenesis, and further studies are needed to confirm the roles of these nine proteins in human ovary in relation to their cell type-specific functions.

The six proteins that displayed discordant mRNA and protein expression patterns (EBLN2, LEKR1, METTL24, TAS1R1, XNDC1N, and ZNF582) should be further validated with orthogonal methods in order to confirm the presence in follicle cells. In addition to challenges based on different age and reproductive status of the samples, variations between mRNA and protein levels could be caused by several factors, including mixed cell clusters in the scRNA-seq data sets or low levels of gene expression in a limited number of cells. It should also be noted that endothelial cell cluster expression levels are merged from various data sets and tissue types in the human body, potentially influencing the results. Interestingly, TAS1R1, also known as Taste 1 Receptor Member 1, displayed pronounced expression in the follicles of the two younger samples (7 and 16 years old), suggesting a potential age-related difference. TAS1R1 has been documented in mammalian spermatozoa but not in oocytes,^20^ and an extended analysis of this MP in relation to reproductive age is of particular interest. Four of the proteins expressed in follicle cells that could not be confirmed by scRNA-seq (EBLN2, LEKR1, METTL24, and ZNF582) were identified also in our previous study. It is noteworthy that genetic variants of LEKR1 have been linked to low birth weight^21,22^ as well as epithelial ovarian cancer,^23^ highlighting this protein as an immensely intriguing candidate warranting further comprehensive investigations.

A limitation with affinity-based proteomics is the access to validated binders, further stressing the need for orthogonal validation. Here, we used antibodies previously validated and optimized within the HPA consortium based on stringent criteria. Since access to representative ovarian samples has been limited in previous experiments using these antibodies, it is however possible that some antibodies may yield nonspecific staining and could be optimized further, or replaced by a more specific antibody. One possibility to increase the signal-to-noise ratio is the use of immunofluorescence. Immunofluorescence also has the advantage of giving the possibility to stain multiple protein targets in the same tissue section, allowing for comparison of the staining patterns with other cell type-specific markers, a strategy that can aid in interpretation of challenging staining patterns. In the present investigation, we utilized this strategy for four proteins (GPR63, TRIM61, TRIM73, and ZNF582) in order to determine if the results observed by IHC could be reproduced by another methodology with the same antibodies but also to investigate if immunofluorescence could improve the specificity. The high consistency between the results generated by IHC and mIF together with more distinct staining observed with immunofluorescence for one of the candidates (TRIM61) suggest that this method could be an important complement to standard IHC in future antibody-based studies analyzing MPs.

Here, we identified 20 MPs expressed in the human ovary and performed an in-depth analysis in four samples corresponding to prepubertal girls and women of fertile age to confirm the expression of these MPs using bulk RNA-seq and IHC of the same tissue samples. The single-cell type-specific expression pattern of 14 proteins could be confirmed by an orthogonal scRNA-seq data set. In spite of the limited characterization of these proteins, we found striking concordance between our results and those derived from transcriptomic analysis. This notable alignment significantly bolsters the credibility and substantiates the validation of these proteins, and we therefore suggest that the data presented here should be sufficient to upgrade the evidence of these proteins to PE1. Both these 14 proteins and the other 6 analyzed proteins constitute interesting targets for further analysis in the context of human ovary using additional samples spanning different stages of the woman’s age, as well as validation with other orthogonal methods, e.g., quantitative methods for protein detection such as mass spectrometry, or spatial transcriptomics analysis.

It should be noted that both sample size and the associated metadata in the present study were limited, potentially affecting the generalizability of our findings. Given that the patients underwent fertility preservation protocols, it is conceivable that they had a history of cancer or other serious illness conditions and potentially underwent treatments prior to ovarian tissue retrieval. While incorporating these data into the HPA database has yielded a valuable enhancement, notably by augmenting follicle counts, it is imperative to underscore this significant limitation. Moreover, due to the scarcity of cells of interest, our analysis in this regard might be constrained. Nevertheless, despite these limitations, our study contributes significantly to the characterization of these proteins in the human ovary and offers promising avenues for future research in the quest of both MPs and further understanding of human reproduction. The transcriptomics data from the four new individuals is publicly available in the HPA database, enabling discovery of new ovary-specific candidates not previously analyzed on the protein level.

While the transition from “Missing Protein” to “PE1″ is well defined and adheres to specific criteria for MS methods,^1^ this is not as clearly established for other approaches, such as those based on antibodies. Antibody-based methods rely on the specificity of binding between antibodies and target proteins, but challenges remain in rigorously validating antibodies and ensuring consistency across results obtained from different techniques. While efforts are made to select highly specific and validated antibodies (as in HPA), the complexity of protein–antibody interactions, variations in antibody affinity and sensitivity, and the diversity of tissues and experimental conditions render antibody-based analysis more prone to ambiguities.

Nevertheless, antibody-based approaches provide the ability to confirm protein localization with a spatial resolution, particularly valuable in tissues where some cells are exceedingly rare, as in this case oocytes. It would appear reasonable that MPs identified through antibody-based proteomics, with confirmation at the transcript level (scRNA), should no longer be classified as MPs. Furthermore, we demonstrate the effectiveness of this approach in identifying the tissues and cell types where missing proteins could be discovered. As a result, these promising candidates could be validated through orthogonal methods, such as targeted mass spectrometry, at the level of specific tissues, even at distinct stages (such as age-related stages in the case of the ovary).

## Supporting Information Available

(Figure S1) Cell-type estimation in fresh frozen histology sections of the human ovary (PDF)
(Table S1) Description of the 61 MPs; (Table S2) description of the 20 final candidates; (Table S3) IHC manual annotation results; and (Table S4) description of mIHC staining protocol and antibody details (XLSX)
