# Supplementary material for: Transcriptomics and Spatial Proteomics for Discovery and Validation of Missing Proteins in the Human Ovary
Source: J Proteome Res. 2023 Dec 12;23(1):238–48. doi: 10.1021/acs.jproteome.3c00545 (PMC10775140; doi:10.1021/acs.jproteome.3c00545)
Supplement: Supplementary file 1 — pr3c00545_si_001.pdf [file pr3c00545_si_001.pdf]

TRANSCRIPTOMICS AND SPATIAL PROTEOMICS FOR DISCOVERY AND VALIDATION OF MISSING PROTEINS IN HUMAN OVARY

*Loren Méar[1,2,3], Xia Hao[4], Feria Hikmet[1], Pauliina Damdimopoulou[2,3], Kenny A. Rodriguez-Wallberg[3,4‡], Cecilia Lindskog[1‡ *]*

‡These authors contributed equally

[1] Department of Immunology, Genetics and Pathology, Cancer Precision Medicine Research Program, Uppsala University, 751 85 Uppsala, Sweden

[2] Division of Obstetrics and Gynecology, Department of Clinical Science, Intervention and Technology, Karolinska Institutet, 14186 Stockholm, Sweden

[3] Department of Gynaecology and Reproductive Medicine, Karolinska University Hospital, 171 77 Stockholm, Sweden

[4] Department of Oncology-Pathology, Laboratory of Translational Fertility Preservation, Karolinska Institutet, BioClinicum, 171 64 Stockholm, Sweden

*** Corresponding Author**

Cecilia Lindskog

Department of Immunology, Genetics and Pathology

Cancer Precision Medicine Research Program

Uppsala University

751 85 Uppsala, Sweden

Email: cecilia.lindskog@igp.uu.se

Supporting Information

S1

Supplementary Figure 1: Cell-type estimation in fresh-frozen histology sections of human ovary

From each fresh-frozen ovary tissue block (ages: 7, 16, 20 and 32), 5 μm thick sections were cut and stained with H&E. Regions of interest (ROI) for follicle-rich, endothelial-rich, and ovarian stroma-rich areas were selected and cells of each type (oocyte, granulosa, endothelial, and ovarian stroma cells) were manually counted. The blow-ups show a magnified image of the cell types indicated below each square and the Count-cells-square shows cells detected with image analysis and manually phenotyped in QuPath software.

Supplementary Figure 1. Cell-type estimation in fresh-frozen histology sections of human ovary from each fresh-frozen ovary tissue block (ages: 7, 16, 20 and 32), 5 μm thick sections were cut and stained with 2H&E. Regions of interest (ROI) for follicle-rich, endothelial-rich, and ovarian stroma-rich areas were selected and cells of each type (oocyte, granulosa, endothelial, and ovarian stroma cells) were manually counted. The blow-ups show a magnified image of the cell types indicated below each square and the Count-cells-square shows cells detected with image analysis and manually phenotyped in QuPath software.

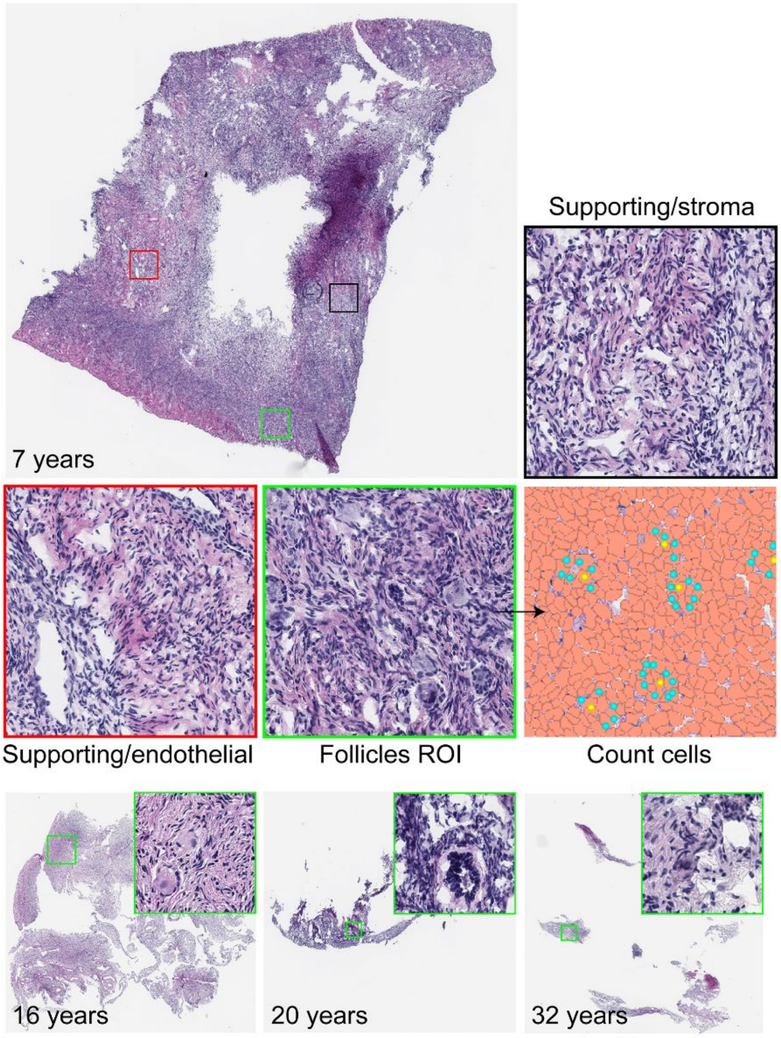

Supplementary Table S1: Description of the 61 MPs

Supplementary Table S2: Description of the 20 final candidates

Supplementary Table S3: IHC manual annotation results

Supplementary Table S4: Description of mIHC staining protocol and antibody details
